# Knowledge of mothers and fathers’ experiences of the early in-home care of premature infants supported by video consultations with a neonatal nurse

**DOI:** 10.1186/s12912-021-00572-9

**Published:** 2021-04-07

**Authors:** Mai-Britt Hägi-Pedersen, Hanne Kronborg, Annelise Norlyk

**Affiliations:** 1 Department of Pediatrics, Slagelse Hospital, 4200 Slagelse, Denmark; 2 Department of Public Health, Faculty of Health, Aarhus University, 8000 Aarhus, Denmark; 3 University College Absalon, Center for Nursing, 4800 Nykoebing F, Denmark

**Keywords:** Experience, Nursing home care, Preterm, Interview, Content analysis

## Abstract

**Aim:**

To gain in-depth knowledge of mothers’ and fathers’ experiences of the whole trajectory of an early in-home care programme supported by video consultations with a neonatal nurse.

**Design:**

A qualitative interview study.

**Methods:**

Data were collected through dyadic semi-structured interviews with mothers and fathers participating in virtual early in-home care programmes and were subjected to inductive content analysis.

**Findings:**

The mothers and fathers were anxious about mastering the care of their premature infants at the start of the early in-home care phase but gradually developed confidence by the completion of the early in-home care programme. Being at home during the early in-home care programme gave the mothers and fathers an opportunity to test their decision making concerning the care of the infant while having the ability to obtain support from nurses when needed.

**Conclusion:**

Our findings indicate that the trajectory of early in-home care programmes combined with video consultations contributes to parents’ increased confidence as mothers and fathers.

**Trial registration:**

Clinical trial registration: REG-113-2014 and SJ-431.

**Supplementary Information:**

The online version contains supplementary material available at 10.1186/s12912-021-00572-9.

## Background

Premature birth, defined as the birth of an infant before the gestational age of 37 weeks [1] is occurring increasingly frequently [2]. In Denmark, approximately 6.4% of all live infants are born prematurely, which amounts to approximately 3700 infants per year [3]. Premature infants need care and treatment in the neonatal ward (NW) until they are physically stable. Many parents experience stress due to hospital admission, which affects the everyday life of the family and results in the separation of mothers and fathers from older siblings [4]. While in the hospital, mothers and fathers gradually take over the care of their infants with support from nurses [5].

In the last phase of hospitalization, most hospitals offer parents in-home care programmes [4–6]. Early in-home care programmes provide an opportunity for infants and parents to go home to continue tube feeding, establish breastfeeding and strengthen family bonding [5]. The participation criteria are usually that the infant is born prematurely, older than 34 weeks’ gestation at the time the family goes home, and physiologically stable and that the parents’ parenting skills are positively assessed by nurses and doctors. Before beginning early in-home care programmes, mothers and fathers are educated about signs of infant illness and the care of premature infants and receive training on placing the nutrition tube and administering first aid at the hospital. During early in-home care programmes, parents are offered support from nurses through either home visits [5, 7, 8] or in-hospital [9] or video consultations [4, 9–11]. The nurse is generally in contact with the parents two to three times a week to provide advice on the infant’s nutrition, the infant’s current weight, and bottle/breastfeeding. Parents can also call neonatal nurses at all hours if questions suddenly arise [9, 10].

Although mothers and fathers experience feelings of happiness to be going home, the shift from hospital to home is also associated with anxiety and insecurity [11]. Furthermore, Flacking et al. [12] emphasize that after leaving the NW, mothers swing between feelings of insecurity, security, exhaustion and relief. Descriptions of fathers’ experiences of returning home are sparse but indicate that fathers are affected by the situation but primarily focus on adapting to the mother’s needs [13]. These observations indicate a need to include both mothers’ and fathers’ experiences in research addressing early in-home care.

As the use of communication technology is growing, video consultations are increasingly offered in early in-home care programmes. However, knowledge is sparse concerning how mothers and fathers experience an early in-home care programme supported by nurse-led video consultations. Studies of early in-home care indicate that video consultations with neonatal nurses can be a supportive practice for parents, as video consultations seem to reduce parents’ feelings of stress [11, 14]. In addition, mothers and fathers seem to feel empowered in the parenting role [14] and feel increased self-efficacy [15] through early in-home care.

The above studies focus on parents’ experiences while at home and indicate that parental participation in an early in-home care programme supported by video consultations may reinforce parents’ ability to master the care of their premature infant.

However, the studies above also illustrate that an early in-home programme includes a whole trajectory from parental training and education at the hospital to mastering the care of the infant at home to parents’ discharge from the programme. Despite the assumed potential of video-supported consultations with a neonatal nurse, knowledge is lacking concerning how mothers and fathers experience the early in-home care programme in its entirety.

Consequently, the aim of this study was to gain in-depth knowledge of mothers’ and fathers’ experiences of the whole trajectory of an early in-home care programme supported by video consultations with a neonatal nurse.

## Methods

This qualitative study is based on semi-structured interviews that were conducted to gain in-depth knowledge of mothers’ and fathers’ experiences of an early in-home care programme with video consultations.

### Context

**Fig. 1:**
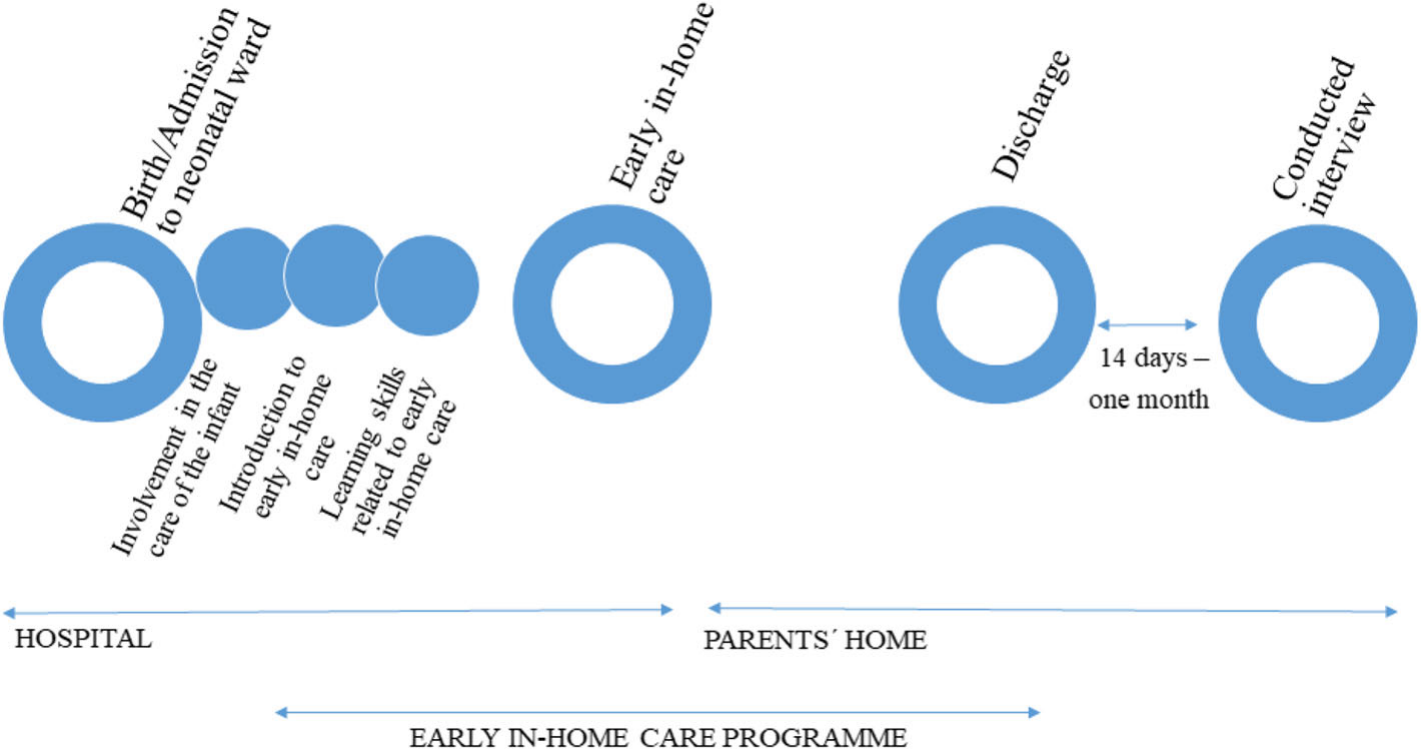
Illustration of the early in-home care programme

Mothers and fathers from two neonatal wards in Denmark offering early in-home care programmes with nurse-supported video consultations participated in the study [9, 10]. The early in-home care programme is illustrated in Fig. 1. To participate in early in-home care, the infants and parents had to fulfil the early in-home care criteria, including a training programme for the mothers and fathers [9].

The parents received support from a team of neonatal nurses and had planned video consultations with the nurses two to three times a week. Furthermore, the parents were offered an infant scale to use at home to weigh the infant prior to the consultations. During the early in-home care, the mothers and fathers controlled infant care, breastfeeding or bottle establishment, and tube feeding at home. The parents and infants were discharged from the early in-home care programme when breastfeeding/bottle-feeding was successfully established and the infants were thriving. Thereafter, the provision of support was taken over by the municipal health care visitor.

The video consultations were performed through the encrypted applications CareRoom and LiveCare provided by ViewCare a/s Søborg. The parents communicated via smartphones, and the nurses communicated via iPads [10].

### Participants

A convenience sample of mothers and fathers with premature infants participating in the programme was recruited from September 2018 to January 2020. The neonatal nurses on the ward informed parents about the purpose of the interview study during early in-home care. Sixteen mothers and fifteen fathers were invited to participate in the study. Eight mothers and eight fathers did not respond, and two mothers and two fathers declined to participate due to time pressure. The six mothers and five fathers who agreed to participate gave consent for the first author to contact them after their completion of early in-home care. The mothers were aged between 21 and 41 years, and the fathers were aged between 24 and 38 years. The infants were born at weeks 27 + 0 to 35 + 5 of gestational age.

### Interviews

To examine both the mothers’ and fathers’ experiences, dyadic interviews were chosen, as they enabled interaction between the parents during the interview; that is, the mothers and fathers were able to supplement each other’s descriptions, elaborate on each other’s expressions, and introduce new themes that deepened the data [16–18].

A semi-structured interview guide was created to gain in-depth knowledge of the mothers’ and fathers’ experiences of the early in-home care programme supported by video consultations with a neonatal nurse. The guide was developed with open-ended questions [19] and contained questions about the parents’ experiences of the early in-home care programme. The open-ended questions were based on previous research of mothers’ and fathers’ experiences of early in-home care programmes [11–13]. Additional questions were -inspired by Bandura’s four sources (mastery experiences, vicarious experiences, verbal persuasion, and emotional and physiological states [20]) to obtain in-depth knowledge of the mothers’ and fathers’ experiences of their capabilities to organize and execute the courses of action required during the early in-home care programme.

The opening question was: *“Please tell me about your experience(s) with early in-home care?”* As Lundqvist et al. [13] indicated that fathers and mothers may experience the transition from hospital to home differently, elaborating questions were addressed to each parent during the interview. Elaborating questions were, for example, *“Can you describe an experience when you experienced joy?”* and *“Can you describe a situation when something went well?”*

The first author (MH), who had been a neonatal nurse for 8 years, carried out the interviews. However, MH was not involved in the care of the infant and had no former relationship with the participants. To enable the mothers and fathers to express their experiences of the trajectory of the early in-home care programme supported by video consultations with a neonatal nurse, the interviews were conducted between 14 days and 1 month after the parents’ discharge from the early in-home care programme; see Fig. 1.

The interviews were performed in the participants’ homes with the infant nearby. The interviews lasted 52–84 min. The audio-recorded interviews were verbally transcribed into 240 pages of text. The transcribed text was transferred to the NVivo 10 software programme, and QSR International helped in organizing the analysis process.

### Data analysis

The interviews were analysed using inductive content analysis as described by Graneheim and Lundman [21]. Inductive content analysis was chosen, as it allowed the identification of patterns to illuminate the mothers’ and fathers’ in-depth experiences. Accordingly, all the interviews were analysed first at the manifest and descriptive levels and then interpreted at the latent level to uncover underlying meanings.

Initially, the transcripts were read several times to obtain a sense of the “wholeness” of the mothers’ and fathers’ experience [21]. Then, the transcriptions were divided into meaning units. The meaning units were condensed and labelled with codes guided by “what the text said” to cover the content of each meaning unit. This process continued through the ordering and grouping of the codes into subcategories via the placement of similar content into broader categories. After the development of each category, the meaning units were read again and compared with the category to ensure consistency.

The next phase involved an expression of the latent and interpretive content of the text, i.e., an interpretation of the underlying meaning [21]. In this phase, we examined “what the text talked about”. The authors discussed the emergent patterns to identify categories representing a thread running through several categories. The analysis process is illustrated in Table 1. Examples from the transcripts are provided to support the findings of the analysis.

**Table 1:** Example of the codes, categories and overarching theme

Theme	Oscillating between feeling confident in caring for the infant on your own and needing support from others				
Category	Becoming comfortable with the idea of early in-home care	Becoming confident in caring for the infant on your own	Becoming confident by having a supportive lifeline	Dealing with social networks –finding a way	Becoming confident in dealing with conflicting advice after losing the lifeline
Codes	Preparation Skills training Tired of the hospital ward Not my infant alone Like the help in the hospital Older siblings	Undisturbed time Freedom Infant scale Using social media New challenges Asking for help or not Open hospital doors	Lifeline Technological advantages Parachute Nurses a call away Friendly push	A dilemma Friends being curious Vulnerable infant Needing practical help	Being discharged Questioning what to do Nurses giving confidence Trusting yourself Conflicting advice

### Ethical considerations

The study was registered and approved by the Danish Protection Agency, Region Sjaelland, Sorø, Denmark, file number REG-113-2014, and approved by the Danish National Committee on Health Research Ethics, Region Sjaelland, Sorø, Denmark, file number SJ-431. The ethical principles of the Declaration of Helsinki were followed [22]. Written informed consent was obtained from the mothers and fathers before their participation. Anonymized transcription and reporting were used to ensure the anonymity of the parents.

## Findings

**Fig. 2:**
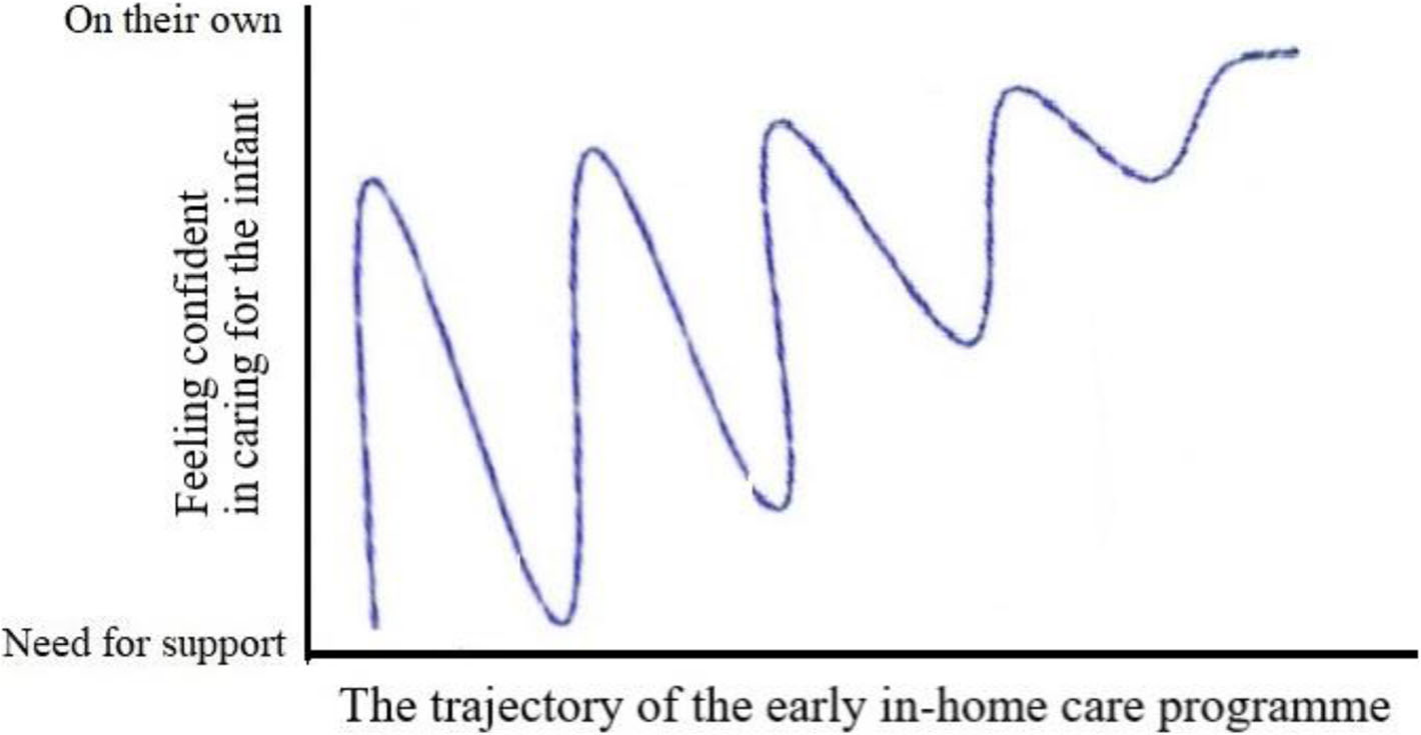
Illustration of the overarching theme of oscillating between feeling confident in caring for the infant on your own and needing support from others, showing the mothers’ and fathers’ development of increased confidence during the trajectory of the early in-home care programme

The analysis of the mothers’ and fathers’ experiences of the trajectory of the early in-home care programme revealed an overarching theme: “Oscillating between feeling confident in caring for the infant on your own and needing support from others”. The oscillation involved a movement towards parental mastery of care with ever-increasing confidence. The overarching theme is illustrated in Fig. 2. This overarching theme refers to the mothers’ and fathers’ ambivalence in both wanting to master the care of their infants on their own but also needing guidance and help from professionals and social networks. The overarching theme is further elaborated by the five categories: “Becoming comfortable with the idea of early in-home care”, “Becoming confident in caring for the infant on your own”, “Becoming confident by having a supportive lifeline”, “Dealing with social networks –finding a way” and “Becoming confident in dealing with conflicting advice after losing the lifeline”.

### Category: becoming comfortable with the idea of early in-home care

In the early phase of admission to the NW, the possibility of an early in-home care programme seemed distant to the mothers and fathers; however, the idea planted a seed of an expectation for the future. The parents began to wonder if such a programme was something that they wanted to do, and they stressed that the experience of not being pushed was important. The parents’ training in parenting skills related to the infant, including the handling of the feeding tube, began the day they were admitted to the NW. This training in practical skills was helpful in making early in-home care seem doable for the mothers and fathers. However, some parents also felt a desire to go home, e.g., because they had older children at home or because they were tired of hospitalization due to repeated room changes caused by new admissions of infants or constant interruptions from the hospital staff.

The parents described a desire to be the primary caregivers. It was difficult for them to be unable to be solely responsible for their own infant in the NW. From the time of the birth, they were eager to learn how to take over the care responsibilities of the nurses. For example, a mother stated, *“... At some point, you just get to where you don’t actually want nurses to touch your child...” (Mother).*

The mothers weighed the pros and cons of leaving the hospital against the benefits of staying there. They could be nervous about needing help from the hospital nurses at home, as illustrated in the following quote from a mother: *“We needed to talk about it for a few days... I don’t think I was as busy coming home as X (the father) because I thought it was really nice with help (from the nurses) … in breastfeeding” (Mother).* The fathers, who often were constantly moving between the NW, work, and home, did not express the same worries about needing help from the nurses at home. The fathers desired freedom regarding logistical issues to have more time with their families. *“I can relax more at home. Without a doubt, I feel better at home” (Father).* Thus, there seemed to be differences between the mothers and fathers concerning the need to enter the early in-home care programme.

### Category: becoming confident in caring for the infant on your own

Being in one’s own home seemed unrealistic and distant to the mothers and fathers for a long time during their stay in the hospital, and therefore finally being at home with the infant seemed too good to be true. One mother illustrated this sentiment by saying, *“It’s a bit like dreaming … like a dream” (Mother).*

The parents compared their experience of the premature birth and the difficulties they had already encountered to the challenges they faced in early in-home care. Accordingly, they perceived the challenges they faced during early in-home care to be less difficult than those they faced at the time of the birth, and they felt increasing confidence to master the challenges on their own. *“I think about everything that has happened. We have done well. And then there’s almost nothing we can’t do now” (Mother).* Furthermore, the mothers and fathers appreciated the freedom to make choices in planning their own day. The daily routines related to the infant and personal care, etc., were similar to those in the hospital, but at home, their everyday life was not dominated by hospital routines, leaving them more undisturbed time to care for their infant. They truly treasured the opportunity provided by the reduced transportation time due to the early in-home care to master the time-consuming challenge of allowing the infant to lie at the breast or have skin-to-skin contact all day if needed. However, travel to the hospital was unavoidable due to both nurses’ desire to see the infant for a check-up and the parents’ feelings of insecurity. *“We had a video meeting with them that day. We talked to Y (the nurse) in there. She says, ‘You have to come because... we have to do something about it...’” (Father).* The prompt assessment and open-door policy at the NW signalled to the mothers and fathers that their difficulties were immediately taken seriously.

However, when alone at home, the parents struggled to decide whether to ask for help. The mothers’ and fathers’ struggles related to questioning whether the infant was thriving and whether anything differed from the previous hour or day. If they doubted their decisions, the parents called the nurse at the NW. The mothers and fathers could also turn to groups for parents of premature infants on social media for answers to questions they had not been able to find elsewhere. They felt increased confidence when other mothers and fathers posted challenges similar to theirs. Consequently, the parents were in constant dialogue with each other about interpreting infants’ signals and what to do next.

Having an infant scale at home was helpful in handling these challenges. The parents used the infant scale as an indicator of whether the infant had gained sufficient weight to gain assurance that the infant was thriving. However, the use of the infant scale at home presented a duality. On the one hand, the scale gave the mothers and fathers certainty and thus contributed to their becoming confident about the infant’s milk intake. As one mother observed, *“It (ed: the infant scale) gave me peace of mind, because then I did not have to worry about how much I thought he was eating now” (Mother).* On the other hand, the infant scale made mothers experience bodily discomfort related to the number on the scale when weighing their infants. *“The uncertainty was overwhelming. My stomach would almost hurt when he was lifted to the infant scale because of the worry of whether he was getting enough to eat” (Mother).* As illustrated, the infant’s weight deeply influenced the well-being of the mother. When the scale showed that the infant’s weight had increased, the bodily feeling of anxiety disappeared immediately. Thus, the infant scale contributed to the mothers’ and fathers’ mastery of their struggles related to their insecurity.

### Category: becoming confident by having a supportive lifeline

Although they desired to be the primary caregivers in the hospital with less interference from the nurses, being at home made the mothers and fathers realize the importance of having a professional partner nearby even if not at home with them. Being at home was described in ways such as *“being thrown out with a parachute”* and *“having a lifeline”*. These descriptions underline that the parents experienced going home as tandem skydiving into the unknown, with the nurses there to assist them with a safe landing.

The mothers and fathers felt confident due to the possibility of going to the hospital or calling the nurses for support if any challenges occurred. This safety net contributed to feeling more relaxed as parents. The experience of being on their own knowing that the nurses were just a phone call away made the parents feel self-reliant. This support increased their belief in their intuition and made them confident in trusting their own decisions. *“We have seen a strength in it (ed: being at home) instead of ‘oh no, now we are alone’. And I think if you have the feeling of being left behind, that we have no help, then I really think it’s going to be uphill” (Father).*

The mothers and fathers were very attentive to the workload of the neonatal nurses and that the nurses were caring for infants who had more serious problems. As a result, the parents felt compelled to resolve some situations themselves to avoid disturbing the NW unnecessarily. However, the parents preferred to call the nurses one time too many if they were insecure, illustrating that the nurses contributed to building up parental confidence through knowledge and guidance.

The mothers and fathers preferred video consultations over ordinary phone calls because of the visual communication between the parents and nurses. They indicated that the video consultations functioned perfectly in terms of sound and picture. The parents referred to the nurses as collaborators, as the nurses could provide professional feedback on the mothers’ and fathers’ questions and worries. The parents related that the nurses trusted them in their role as caregivers. *“To talk to someone (ed: nurse) who takes you very seriously … when you have talked to the nurse, it’s like you’re thinking, ‘Well yeah, it wasn’t totally off what I had considered’”* (Mother). The parents were uncertain of the next step(s), and they therefore embraced the guidance they received from the nurses, which the parents referred to as *“a friendly push”.* Thus, the mothers and fathers felt empowered in interpreting the infants’ signals and caring for the infants.

### Category: dealing with social networks–finding a way

During early in-home care, the mothers and fathers tried to find a way to interact with their social networks. Social networks generated positive feelings, such as comfort and support, and helped the parents reflect on their parenting role but also created many difficult feelings, such as anger, sadness and disagreement.

Being at home with the premature infant created, however, a dilemma concerning support from close relatives. On the one hand, the mothers and fathers highlighted that it was important to be on their own and not be overrun by visits. On the other hand, the mothers and fathers needed practical help from relatives and felt supported when they were praised for doing well in their role parenting a premature infant.

It was difficult for close relatives to understand that the premature infant was more vulnerable than other infants and that the infant might not act like a full-term infant. The parents expended much effort during early in-home care to make others, e.g., the infants’ grandparents, understand the precautions that they, as parents, took to protect their infants. *“I can understand that it is hard for them that we choose not to see our close family. All they see is a happy boy. We can feel the difference when there is someone other than us; he is different and tense” (Mother).*

In contrast to close family, the mothers and fathers found friends more likely to be curious and open-minded about their situation. Their friends had often recently had children themselves, making them aware of the worries and difficulties that the care of an infant created. The parents could draw on challenges faced by their friends to reflect on their own situations. Furthermore, they could embrace their friends’ advice or discuss their worries with them, as the mothers and fathers found their friends less judgemental than close family.

### Category: becoming confident in dealing with conflicting advice after losing the lifeline

After discharge from the early in-home care programme, the parents realized that they were on their own, as the easily accessible lifeline of the nurses on the NW was gone. Simultaneously, however, they felt relieved about finally being discharged and relying on assistance from the municipal health department like parents with full-term infants. This change signalled normality.

The mothers and fathers described the shift from the early in-home care programme with neonatal nurse support to assistance from the municipal health visitor as *“seeing the light at the end of the tunnel”*, *“the infant finally being ‘a real infant’”* and “*everything ‘finally being normal’”.* This allowed the parents to become closer to realizing their expectations of a normal family life with their premature infant.

The loss of easy contact with neonatal nurses, however, made the parents rely on advice from new professionals, such as municipal health visitors or general practitioners. Often, the new advice differed from the knowledge they acquired during hospitalization and early in-home care programmes. One father explained, *“We’ve mixed a little bit of the two things we’ve been recommended... we’ve taken 50% from the neonatal ward and 50% from the municipal health visitor, and then we made our own mix of what we think fits” (Father).*

Consequently, the mothers and fathers found that the early in-home care programme gave them the confidence to pick and choose between different pieces of advice and strengthen their decision making related to the care of their premature infant.

## Discussion

The findings illustrate that the trajectory of the early in-home care programme supported by video consultations with a neonatal nurse contributed to the mothers and fathers becoming confident in mastering caring for their premature infant on their own. The overarching theme “Oscillating between feeling confident in caring for the infant on your own and needing support from others”, however, revealed the parents’ ambiguity in both wanting to master the care of their infants and needing advice from professionals and practical help from social networks.

The parents expressed two main issues related to their experiences of being at home. It was pleasant for the parents to simply be at home as a family in their own sphere. The other issue concerned the mothers and fathers resolving challenges related to the infant on their own, albeit with access to help from the nurses in the NW.

Our study differs greatly from previous analyses of early in-home care, as the purpose of those studies was to assess the experience of mothers and fathers during the phase in which they were at home [14, 23]. Our findings demonstrate that during the whole trajectory of the early in-home care programme, the parents gradually became self-reliant in making decisions about the care of their premature infant as they became increasingly confident. As a result, their experiences of early in-home care with nurse-supported video consultations were positive. In contrast, Holm et al. [14] reported that parental confidence primarily evolved at home during early in-home care. Our study showed that the mothers and fathers perceived that the nurses had time to help them, were supportive, and provided knowledge that helped them become confident as parents, consistent with the findings of Holm et al. [14] and Lindberg [23].

One significant finding was that the early in-home care was gradually introduced to the parents by continuously expanding the knowledge, training and education provided by the nurses. This made early in-home care a natural extension of hospitalization. This finding is supported by Brodsgaard et al. [5], who state that with an introduction to the idea of early in-home care, through time and teaching, the parents become confident and prepared for it. Our study adds to these findings by showing that the mothers’ and fathers’ decision to go home was closely connected to the desire for the family to be together at home as also indicated by Lundqvist et al. [13]. Unlike Wigert et al. [24], we did not find that the parents felt that the staff had unspoken expectations during the hospital stay, making the parents feel insecure in their parenting roles and that they were bearing unwanted responsibility. However, our findings illustrated that early in-home care may be seen as parents’ response to hospitalization and as parents’ perception of a possible way to leave the hospital and be together at home. Our study indicated that the mothers’ and fathers’ purposes for entering the programme differed. According to findings by Hemle Jerntorp et al. [25], fathers prioritized the mother, thus ignoring their own needs during admission, but fathers felt more involved in the care of the infant during early in-home care [25]. Additionally, our findings suggest that early in-home care promoted the involvement of the father in caring for the premature infant.

Another important finding was the significance of having easy access to communication with nurses through video consultation. This finding is similar to that of Holm et al. [14]. However, unlike Holm et al. [14], we found that an important factor for the parents was that the video consultations saved time that could be spent as important quality time with the infant, as the video consultations did not require time for transportation. This finding is line with Lindberg [23], who found that video consultations were especially important when families lived a long distance from the hospital. While our findings demonstrated that physical check-ups of infants in the hospital are unavoidable, whether initiated by the parents themselves or requested by NW nurses, the mothers and fathers welcomed those hospital check-ups. Earlier studies also showed that unplanned consultations and physical check-ups of infants in the hospital are unavoidable [9, 10]. Lindberg et al. [11] stressed that parents view video consultations primarily as a supplement that could not replace physical consultations. Our findings, similarly, indicate that video consultations alone are not currently sufficient to support parents caring for infants, suggesting a need to continuously focus on developing the technology and its opportunities.

Our findings further indicated that the mothers and fathers, although relying on support from nurses nearby, mostly wanted to solve the problems they faced on their own. According to Dellenmark-Blom and Wigert [6], parents become empowered to be primary caregivers and have the nurse serve the needs of the family. The process of becoming confident occurred according to our findings throughout hospitalization and during the early in-home care programme with accessible support from the nurses. The parents developed confidence in the idea of early in-home care, confidence in caring for the infant on their own by having a supportive lifeline of nurses, confidence in dealing with social networks and confidence in dealing with conflicting advice. Thus, our findings indicate that the early in-home care programme allowed the mothers and fathers to gradually increase their control over the infants’ care in a positive way. This finding suggests that the early in-home care made the fathers and mothers a cohesive couple who cared for their infants together and allowed the fathers to have more involvement in family life, as found by Hemle Jerntorp [25].

Our findings also indicated a paradox. Although the parents sometimes struggled with feelings of insecurity and felt a need for support and guidance from nurses, they hesitated to call for fear of disturbing the nurses. Ericson et al. [26] defined “reactive support” as a request for support wherein parents wait but then ultimately call nurses with a question, and they identified a duality in this reactive support. Mothers were grateful to have a telephone number to call if they needed support but also had difficulty deciding whether their problem was serious enough to warrant making a call. Neill et al. [27] reported that parents’ anxiety to ask for help could potentially result in reduced or inappropriate information for the mothers and fathers concerned. Wigert et al. [24] stated that parents find it difficult to request information from health professionals because parents do not know what questions to ask. Consequently, parents may have unresolved issues despite the easy access to support from nurses. This indecision about calling the nurses might explain why some mothers and fathers in our study turned to social media or social networks for answers. Danbjorg et al. [28] reported that parents used asynchronous online chats to ask nurses questions rather than calling the nurses directly on the telephone. Together with our findings, this might indicate that mothers and fathers would benefit from video consultations supplemented with online chat or forums in early in-home programmes. This possibility requires further exploration.

### Study limitations

One limitation of this study was that the participants reflect a select group due to the inclusion criteria of the early in-home care programme. However, the intention of this study was not to generalize about mothers’ and fathers’ experiences but to advance the understanding of their experiences of the trajectory of the early in-home care programme with nurse-supported video consultations.

The use of dyadic interviews enabled interactions between the parents, and it seemed that the mothers and fathers supported and elaborated on each other’s experiences during the interviews. However, there might be a risk of parents withholding some experiences when their partner is present [18]. Furthermore, one couple was interviewed without the father, which may have limited the nuances of their shared experiences.

The study was based on semi-structured interviews and open-ended questions. Throughout the interviews, the opening questions allowed the parents to tell their stories, and the additional questions allowed us to examine the mothers’ and fathers’ experiences related to their ability to master the care of the premature infant. However, the additional questions were inspired by Bandura’s four sources, and this framework might have limited some parents’ elaboration of their experiences.

## Conclusions

This study explores how mothers and fathers experience the trajectory of an early in-home care programme and illustrates the process by which the early in-home care programme with video consultation support contributes to parents becoming confident in mastering the care of their premature infant. Mothers’ and fathers’ experiences of the trajectory of the programme revealed ambiguity in wanting to master the care of their infant while also needing guidance and help from professionals and social networks. Our findings show that the parents were nervous about mastering the care of their premature infants at the start of the early in-home care phase but gradually developed confidence by the completion of the early in-home care programme with nurse-supported video consultations.

Consequently, our study indicates that being at home during the early in-home care programme gives parents an opportunity to test their decision making concerning the care of the infant while having the ability to turn to support from the nurses when needed.

## Implications

This study showed that early in-home care with video consultations for premature infants and parents supported mothers and fathers in becoming confident in mastering the care of their premature infants. Based on our findings, we recommend the following:

- An increased focus on adapting the technology to the needs of individual parents, e.g., by providing asynchronous support, is needed. Furthermore, an increased focus on training nurses in the use of video consultations as a form of communication is needed.
- An increased focus on nurses being more proactive, e.g., calling parents themselves, giving the mothers and fathers an opportunity to ask questions they may have, or offering to involve grandparents in their communication.

## Supplementary Information

**Additional file 1.** Interview guide.

## Data Availability

The datasets used and analysed during the current study are available from the corresponding author on reasonable request.
