# Supplementary material for: Knowledge of mothers and fathers’ experiences of the early in-home care of premature infants supported by video consultations with a neonatal nurse
Source: BMC Nurs. 2021 Apr 7;20:54. doi: 10.1186/s12912-021-00572-9 (PMC8028708; doi:10.1186/s12912-021-00572-9)
Supplement: Supplementary file 1 — Additional file 1. Interview guide. [file 12912_2021_572_MOESM1_ESM.docx]

**Interview guide**

*Opening question:*

Will you please tell me about your experience(s) with early in-home care?

*Elaborating questions:*

Mastery Experiences:

Can you describe an experience when you experienced joy?

Can you describe a situation when something went well?

Physiological and emotional states:

Will you please describe what it has meant to participate in early in-home care? For you as a mother? For you as a father?

Will you please tell me about an experience where you felt insecure?

What have you done when you have felt insecure?

Vicarious experiences:

Have you heard about experiences from others related to caring for your infant?

Verbal persuasions:

How did you experience support from the health professionals, social networks and family?

How did you communicate with the neonatal section?

*The interviews were in Danish, so these questions represent a translated version.*
